# Wellbeing in Urban Greenery: The Role of Naturalness and Place Identity

**DOI:** 10.3389/fpsyg.2018.00491

**Published:** 2018-04-11

**Authors:** Igor Knez, Åsa Ode Sang, Bengt Gunnarsson, Marcus Hedblom

**Affiliations:** ^1^Department of Social Work and Psychology, Gävle University College, Gävle, Sweden; ^2^Landscape Architecture, Planning and Management, Swedish University of Agricultural Sciences, Uppsala, Sweden; ^3^Department of Biological & Environmental Sciences, University of Gothenburg, Gothenburg, Sweden; ^4^Department of Forest Resource Management, Swedish University of Agricultural Sciences, Uppsala, Sweden; ^5^Department of Ecology, Swedish University of Agricultural Sciences, Uppsala, Sweden

**Keywords:** naturalness, urban greenery, place identity, wellbeing, affect-regulation

## Abstract

The aim was to investigate effects of urban greenery (high vs. low naturalness) on place identity and wellbeing, and the links between place identity and wellbeing. It was shown that participants (Gothenburg, Sweden, *N* = 1347) estimated a stronger attachment/closeness/belonging (emotional component of place-identity), and more remembrance and thinking about and mental travel (cognitive component of place-identity) in relation to high vs. low perceived naturalness. High naturalness was also reported to generate higher wellbeing in participants than low naturalness. Furthermore, place identity was shown to predict participants’ wellbeing in urban greenery, accounting for 35% of variance explained by the regression. However, there was a stronger relationship between the emotional vs. the cognitive component of place identity and wellbeing. Finally, a significant role of place identity in mediating the naturalness-wellbeing relationship was shown, indicating that the naturalness-wellbeing connection can be partly accounted for by the psychological mechanisms of people-place bonding.

## Introduction

People form ties with physical places including psychological, social, historical, religious, health, and cultural connotations (Graumann, 2002; Knez, 2005; Lewicka, 2008; Knez et al., 2009; Lachowycz and Jones, 2013). Accordingly, places can act as reminders of important personal and collective experiences and identifications (Knez, 2006; Lewicka, 2008; Wang, 2008; Taylor, 2010; Wheeler, 2014) comprising different emotions, cognitions, behaviors, and traditions in how we perceive and comprehend surroundings and ourselves. Places, in other words, serve to position the psychological self (Canter, 1997; Casey, 2000; Knez and Thorsson, 2006; Knez, 2014) operating on personal and collective experiences and resulting in different types of identifications (Twigger-Ross et al., 2003; Wang, 2008; Stobbelaar and Pedroli, 2011; Clayton, 2012; Wheeler, 2014). (See also Neisser, 1988 and Leary and Tangney, 2003 for a discussion about different types of self- and identity-constructions in psychology.)

### Place Identity

Previous theoretical accounts on person-place bonding have suggested concepts of sense of place, place attachment, and place identity (Jorgensen and Stedman, 2001, 2006; Brown and Raymond, 2007; Droseltis and Vignoles, 2010; Scannell and Gifford, 2010). The type of people-place bonding of primary interest here is the personal identification with a physical place (place identity) involving place-related knowledge and feelings apportioned across declarative memory as autobiographical memory (Kihlstrom and Klein, 1994; Conway, 2005).

The function of this type of memory is to ground the self and its social position, as well as to regulate current and future behaviors, problems, and goals (Adler, 1931; Neisser, 1988; Singer and Salovey, 1993; Pillemer, 2003; Conway, 2005; Knez, 2017; Knez and Nordhall, 2017; Knez et al., 2017). Autobiographical memory is consciously experienced as a narrative; as “my life story” (Fivush, 2008) including several context-specific selves, identities (McConnell, 2011; Knez, 2016). One such self/identity is a place identity. It involves place-related recollections of “perceptual, semantic, and emotional characters of periods of our lives” (Knez, 2006, p. 359).

In line with, for example, classical identity theory suggesting processes of emotion and cognition in identity formation (e.g., Tajfel, 1972, 1978; Hogg, 2012), Knez (2014) suggested a role for two psychological components accounting for the place identity. A cognitive component including processes of mental temporality (inner “time travel”), coherence, correspondence, reflection, and agency (see Conway et al., 2004; Klein et al., 2004), and an emotional component involving the process of attachment/closeness/belonging (e.g., Marris, 1982; Hidalgo and Hernandez, 2001; Giuliani, 2003; Knez, 2005). This suggests that we do not only think, remember and reason (cognitive component) about places in our life but we also emotionally attach (emotion component) to these places. In the words of Knez (2014, p. 186): “the physical places and time *position*-anchor one’s reminiscence by forming psychological person-place ties, emotional and cognitive bonds that conduct the psychological agent toward physical place and time as the organizing formats for its personal memory.”

It is furthermore shown that a place identity comprises nature-related qualities, even local climate details (Knez, 2005) such as “cold clear air” and “burning hot sun” (Knez, 2006). In line with this, residents living in a mountainous county have recently been shown to have a strong place identity with the surrounding nature (Knez and Eliasson, 2017), suggesting that nature might be part of a person’s life-story (Fivush, 2008; Knez, 2014); leading to, for example, feelings of emotional loss after a natural disaster (Knez et al., 2018). Knez and Eliasson (2017) showed furthermore that when visiting these natural sites respondents perceived, in a self-regulating way (Korpela, 1989, 1992; Korpela and Hartig, 1996; Knez, 2006), higher levels of wellbeing. This indicates that a naturalness-wellbeing (Maller et al., 2005; Carrus et al., 2015b) connection might be accounted for by the psychological mechanisms of people-place bonding; implying a *mediation role* of place identity in the nature wellbeing relationship.

### Benefits of Exposure to Nature

In many cultures archetypical types of nature-related places have been associated with the finest types of living (Ward Thompson, 2011). Restorative potential of nature has been reflected upon for a long time (Linné, 1811; Gesler, 2000) as well as suggested to involve an innate tendency of humans to seek connections with nature (Wilson, 1984; Kellert and Wilson, 1993). In line with this, nature-related settings have been indicated to associate with positive affect, feelings of solitude and aesthetical values and a sense of timelessness (Laski, 1961; Williams and Harvey, 2001; Park et al., 2011; Russell, 2012), as well as with physiological, psychological, and social variables (Abraham et al., 2010; Bowler et al., 2010; Hartig et al., 2011; Carrus et al., 2015b; Sandifer et al., 2015). These types of findings have been related to the urban greenery too (e.g., Carrus et al., 2013, 2015a, 2017; Panno et al., 2017); however, also including the ambivalence of attitudes toward the urban greenery as such (Bonnes et al., 2004, 2011; Van den Berg and Konijnendijk, 2012).

Most of these data have been theoretically framed within emotional, aesthetical, and cognitive aspects of nature-wellbeing relationships (Ulrich, 1983; Korpela, 1989; Kaplan, 1995; Scannell and Gifford, 2010; Hartig et al., 2011; Clayton, 2012; Pretty et al., 2015). However, relationships between the phenomena of place-related identity, memory, and wellbeing have been sparsely addressed. Ratcliffe and Korpela (2016), Knez and Eliasson (2017), and Morton et al. (2017) have recently indicated several types of relationships between the psychological processes of identity and memory and restorative potentials of nature.

### Present Study

As shown above, previous research has reported health and wellbeing benefits of the natural environment for humans (Abraham et al., 2010; Bowler et al., 2010; Hartig et al., 2011; Lachowycz and Jones, 2013; Bratman et al., 2015; Carrus et al., 2015b; Sandifer et al., 2015). In addition, Knez and Eliasson (2017) reported a positive relationship between persons’ nature-related place identity and wellbeing implying a mediation role for place identity in the nature-wellbeing link. Our general objective was to broaden Knez and Eliasson’s (2017) mountain landscape-related results to include urban natural milieus, and to test the hypothesis that an influence of naturalness on wellbeing is mediated by place identity: nature (predictor) → place identity (mediator) → wellbeing (criterion).

Urbanization is increasing across the globe, a process that will intensify the pressure on urban greenery (James et al., 2009). This type of environment plays a significant role in sustainable development (Pauleit, 2003; Konijnendijk et al., 2013) and human wellbeing (Lachowycz and Jones, 2013; Haase et al., 2014; Van den Berg et al., 2014). Previous studies have, for example, shown that natural, compared to non-natural, settings might be more effective in stress recovery (Hartig et al., 1991; Purcell et al., 2001; Staats et al., 2003; Tyrväinen et al., 2014; Van den Berg et al., 2014) and that the degree of naturalness (Ode Sang et al., 2008) might be related to greenery preferences (Ode Sang et al., 2009; Van der Jagt et al., 2014; Junge et al., 2015). Furthermore, Ode Sang et al. (2016), Gunnarsson et al. (2017), and Hedblom et al. (2017) have recently shown that people value urban greenery significantly more when it includes high rather than low biodiversity, and that naturalness generates well-being for residents living close to urban green spaces.

In line with previous findings on the positive relationships between nature and wellbeing (Millennium Ecosystem Assessment [MEA], 2005; Abraham et al., 2010; Bowler et al., 2010; Hartig et al., 2011; Carrus et al., 2013; Sandifer et al., 2015) and the role of identity and memory in nature-related restoration (Ratcliffe and Korpela, 2016; Knez and Eliasson, 2017; Morton et al., 2017), we investigated: (1) Effects of type of urban greenery (high vs. low perceived naturalness) on place identity and wellbeing; (2) Connections between place identity and wellbeing; and (3) The mediating role of place identity in the link between naturalness and wellbeing.

We assumed that urban as well as rural greenery might be part of a person’s identity (Borrie and Birzell, 2001), intertwined with the place-related self (Tuan, 1977; McConnell, 2011; Knez, 2014). Accordingly, people may revisit urban greenery in a self-regulating way (Korpela, 1989, 1992; Korpela and Hartig, 1996; Parkinson and Totterdell, 1999; Knez, 2006) in order to increase wellbeing (Tzoulas et al., 2007; Tyrväinen et al., 2014; Carrus et al., 2015a). According to the theory of self-regulation, we do control and adjust our behaviors and moods proactively by telling ourselves to minimize negative behaviors and moods (Carver and Scheier, 1990; Bandura, 1991; Heatherton, 2011; Mann et al., 2013).

### Hypotheses

Accordingly and in line with previous research (e.g., Korpela, 1989; Knez, 2006; Hartig et al., 2011; Carrus et al., 2013; Ratcliffe and Korpela, 2016; Knez and Eliasson, 2017), we predicted that high naturalness will have positive effects on wellbeing and on emotional and cognitive components of place identity (*hypothesis 1*), a positive association between the components of place identity and wellbeing (*hypothesis* 2), and a mediating role of place identity in the naturalness-wellbeing relationship (*hypothesis 3*). Finally, we assumed that the emotion-wellbeing compared to the cognition-wellbeing relationship would be stronger (see *hypothesis 2*), because emotion may: (1) Boost autobiographical memory processes (Canli et al., 2000; Phelps, 2006); (2) Control/adjust psychological processes (Gross, 2010); and (3) Precede cognition in people-place bonding (suggesting that emotional compared to cognitive component might be more boosted in place-identity; Knez, 2014).

## Materials and Methods

### Sample

Gothenburg is the second largest city in Sweden. Ca 500,000 people live on an area of 448 km^2^ (57°42′N, 11°58′E). The city will have 150,000 more residents by the year 2035 and that the metropolitan area is expected to reach 1 million in 2017. A postal survey was sent to a total of 2866 Gothenburg households living close to six different urban green spaces. They were chosen at random from a population register. The survey comprised several sections, with questions about demographic variables and people’s experiences, activities, perceptions, and attitudes toward green spaces. After three contacts with prospective respondents, a total of 1347 replies were obtained. Fifty-six point eight percent of respondents were women and 43.2% men, distributed across six age groups, ≤25 (9.2%), 26–35 (24%), 36–45 (12.5%), 46–55 (14.4%), 55–65 (21.4%), and 66+ (18.3%).

The survey was conducted in accordance with APA’s (American Psychological Association) ethics code. Accordingly, participants were informed (written consent) about: (1) the aim of our research, its procedures, benefits to society and especially to people living in a city nearby urban greenery, as well as the length of participation; (2) their right to withdraw from the study at any time without any consequences; (3) reasonable factors that may influence their willingness to participate, for example, how long it will take to complete the questionnaire and information about the types of questions included in the questionnaire; (4) confidentiality; (5); that they will not be financially compensated for participation; (6) whom to contact about any questions related to the study. We mentioned also that findings based on this survey will be reported in multiple publications (see Ode Sang et al., 2016; Hedblom et al., 2017; Gunnarsson et al., 2017 for previous publications).

### Settings

**FIGURE 1:**
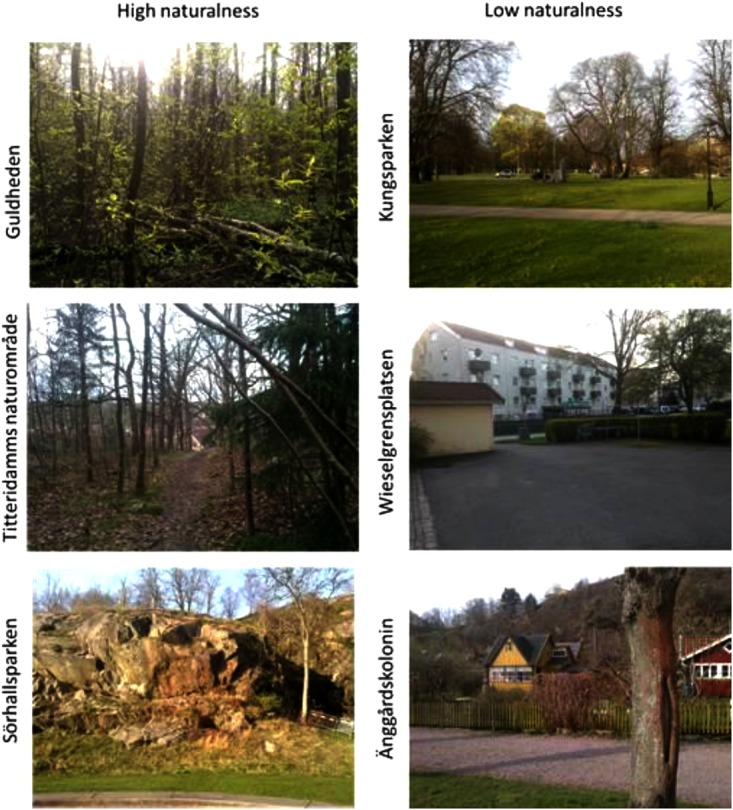
Photos (by Erik Heyman) of the six different settings (from a previous open access publication, Hedblom et al., 2017) representing high vs. low naturalness.

The six green spaces were located across the city. They represented different types of green space (Guldheden, Kungsparken, Sörhallsparken, Titteridamm, Wieselgrensplatsen, and Änggårdens kolonier; see **Figure 1**). The common denominators for all six areas are that they are publicly accessible and integrated into existing residential areas, and hence used by local residents. Spaces were redefined to two naturalness categories of high versus low, based on the *mean value* of the perceptual classifications/scales of “nature like” and “wild.” These were estimated on a 7-point Likert scale by respondents living nearby each respective green space. More precisely, the mean value of 4.25 represented the *cut-off point* for the high vs. low naturalness categories (see **Table 1**). This was done in line with Tveit et al. (2006) and Ode Sang et al. (2009) framework for analyzing visual landscape dispositions. As can be seen in **Table 1** and **Figure 1**, Guldheden, Sörhallsparken, and Titteridamm were defined as areas of high perceived naturalness; Kungsparken, Wieselgrensplatsen, and Änggårdskolonin as areas of low perceived naturalness.

**Table 1:** Mean estimations of the perceptual categories of nature-like and wild for the six settings, respectively, defining the two categories of high vs. low naturalness.

Settings	Nature-like	Wild	*M*	High/low naturalness
Guldheden	5.59	4.64	5.12	high
Kungsparken	4.19	2.75	3.47	low
Sörhallsparken	4.93	3.68	4.31	high
Titteridamm	5.6	4.63	5.12	high
Wieselgrensplatsen	3.99	2.82	3.41	low
Änggårdskolonin	4.72	3.43	4.08	low

**Guldheden** is a centrally located urban woodland that is surrounded by local traffic routes, residential areas with tall tower blocks and three-story buildings, and a University hospital. The vegetation is dominated by deciduous trees, e.g., English oak (*Quercus robur*) and birch (*Betula pendula, Betula pubescens*). There are a few walkways through the area. The maintenance of the woodland is minimal.
**Kungsparken** is an old formal park (ca 150 years) in the city centre. It is surrounded by multi-story residential buildings from the late 19th century and a canal from the 17th century. Multiple busy traffic routes are crossing the park, as well as a number of walkways. Veteran trees, e.g., English oak, lime (*Tilia cordata*) and beech (*Fagus sylvatica*), are predominant in the area. The ground is covered by large, mown lawns.
**Sörhallsparken** is a combination of a recently established, formal park and an urban woodland on a rocky hill in the middle. The park is surrounded by newly built row houses and multi-story residential buildings, and the Göta River. The new park is dominated by large lawns and a few ornamental trees. The vegetation of the woodland is predominantly English oak, birch and Swedish whitebeam (*Sorbus intermedia*).
**Titteridamm** is a suburban woodland that is surrounded by traffic routes and residential areas with row houses. The vegetation in this nature area is a mixed forest with Scots pine (*Pinus sylvestris*), Norway spruce (*Picea abies*) and birch as predominant tree species. Apart from a small area with a pond, the maintenance of the woodland is minimal. There are a few trails through the area.
**Wieselgrensplatsen** is a residential area with three-story buildings from 1940s. Large well managed lawns dominate the ground between the buildings. A few trees and some ornamental plants are found in the courtyards. Some local roads are crossing the area.
**Änggårdens** kolonier is an old allotment area (ca 100 years) that consists of ca 50 small private gardens with cottages and a common lawn. Public walkways allow people to pass through the area which lies between a campus area (with a medical faculty and biology) and residential three-story buildings. The vegetation is mainly domesticated trees and plants, e.g., apple (*Malus x domestica*), black and red currants (*Ribes* spp.), and multiple ornamental plants.

### Measures

#### Place-Identity

This measure involves an autobiographical emotional and a cognitive component comprising ten statements (see Knez, 2014; Knez and Eliasson, 2017). *Emotional component* (processes of attachment/closeness/belonging; in the present study, with a Cronbach alpha of 0.89): “I am keenly familiar with the place.”; “I miss it when I’m not there.’; “I have strong ties to the place.”; “I am proud of the place.”; “The place is a part of me.”. *Cognitive component* (processes of coherence, correspondence, mental temporality, reflection, and agency; in the present study, with a Cronbach alpha of 0.93): “I have had a personal contact with this place over a long period.”; “There is a link between the place and my current life.”; “I can travel back and forth in time mentally to this place when I think about it.”; “I can reflect on the memories attached to this place.”; “These thoughts about the place are part of me.”. Participants were asked to respond to these statements on a 7-point scale ranging from 1 (completely disagree) to 7 (completely agree).

#### Wellbeing

Participants were asked to respond to ten statements from “The World Health Organization (10) well-being index” (Bech et al., 1996), measuring their place-related well-being. They responded to the question of *when I’m on the site, I feel*: “Sad and down”; “Calm and relaxed”; “Energetic, active, and enterprising”; “Relaxed and refreshed”; “Happy and pleased with my personal life”; “Satisfied with my living situation”; “I live the life I want to live”; “Inspired to deal with today’s work”; “I can cope with serious problems or changes in my life”; “That life is full of interesting things.” The 4-point scale from the original measure was replaced (Knez and Eliasson, 2017) by a 7-point scale, ranging from 1 (completely disagree) to 7 (completely agree), with a Cronbach alpha of 0.93.

### Design and Analyses

We used a non-equivalent comparison-group quasi-experimental design (McGuigan, 1983) for the *hypothesis 1* analyses (see Introduction section). Accordingly, unlike a ‘true experiment’ (Liebert and Liebert, 1995), the causal inferences drawn from these types of results are considered to be weaker. A MANOVA was used for the dependent variable place identity (two measures), and an ANOVA for the dependent variable well-being (one measure), involving the between-subject independent variable naturalness (high vs. low). The association between place identity and wellbeing (*hypothesis 2* analysis) was calculated with a multiple linear regression analysis defining the two (emotion and cognition) components of place identity as predictors and wellbeing as the criterion variable. A mediating role of place identity (including emotion and cognition components as an index) in naturalness-wellbeing relationship (*hypothesis 3*) was also investigated by performing a mediation analysis, using the plug-in PROCESS (e.g., Hayes, 2013) developed for IBM SPSS Statistics.

## Results

First we report the effect of naturalness on place identity and wellbeing (variance analyses), second the links between place identity and wellbeing (regression analysis), and third the mediating role of place identity in the naturalness-wellbeing link.

### Effects of Naturalness on Place Identity and Wellbeing

**FIGURE 2:**
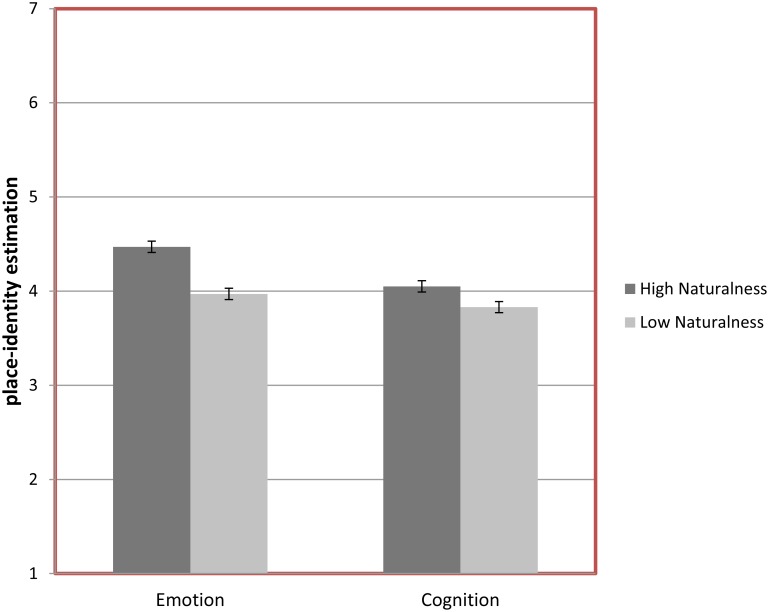
Mean emotion and cognition in place identity as a function of high vs. low naturalness in urban greenery.

A MANOVA showed a main effect of naturalness on place identity, Wilks’s λ = 0.97 (2, 1303) = 21.53, *p* < 0.01, η^2^ = 0.03, associated with emotional (*p* < 0.01) and cognitive (*p* < 0.05) components of place-identity. As can be seen in **Figure 2**, high naturalness was shown to generate more emotions (attachment/closeness/belonging) and cognitions (remembrance, thinking, and mental travel) than low naturalness.

A main effect (ANOVA) of naturalness on wellbeing, *F*(1,1276) = 9.19, *p* < 0.01, η^2^ = 0.01, showed that high compared to low naturalness generated more wellbeing (*M* = 4.64, *SD* = 1.25 vs. *M* = 4.43, *SD* = 1.24).

### Association Between Place Identity and Wellbeing

According to the regression analysis, both components of place identity were shown to significantly predict wellbeing, accounting for 35% of variance explained (see **Table 2**). However, as can be seen in **Table 1**, the emotional compared to cognitive component of place identity was shown to have a higher beta coefficient value, indicating a higher increase in wellbeing for every 1-unit increase in emotional vs. cognitive component of place identity.

**Table 2:** Regression statistics for the relation between place identity (emotion and cognition components) and wellbeing.

*R*^2^	Beta	SE	df	MS	*F*	*t*	Significance
0.35			2,1266	339.6	334.56		0.00
0.49	(emotion)	0.03				14.07	0.00
0.12	(cognition)	0.02				3.41	0.00

### Mediating Role of Place Identity in Naturalness-Wellbeing Link

Since naturalness had a significant effect on wellbeing and on both components of place identity (see section “Effects of Naturalness on Place Identity and Wellbeing”), and there was a significant positive association between place identity and wellbeing (see section “Association Between Place Identity and Wellbeing”), a test for the mediating role of place identity (index level of place identity comprising both components; mediation analyses including emotion and cognition components of place identity, respectively, as mediator were also performed, see *Addendum* below) in naturalness-wellbeing relationship was conducted. A mediation analysis PROCESS developed by Hayes (2013) for IBM SPSS was performed.

**FIGURE 3:**
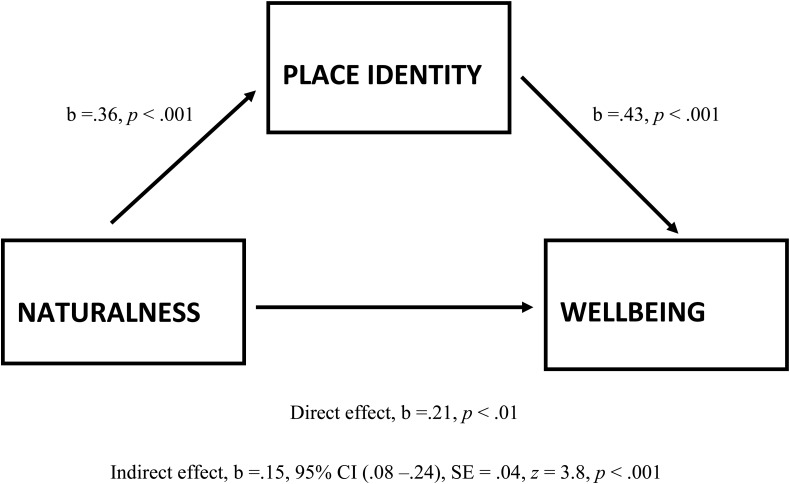
Mediation model of naturalness as a predictor of wellbeing, mediated by place identity, including the mediation analysis statistics for relationships of: (1) naturalness → place identity; (2) place identity → wellbeing; (3) naturalness → wellbeing (direct effect); and (4) naturalness → wellbeing via place identity (indirect effect = mediation). (*b* is an unstandardized regression coefficient, and CI is confidence interval for the bootstrap methods, between BootLLCI and BootULCI).

The results showed (see **Figure 3**) that: (1) naturalness predicts place identity (*b* = 0.36, *p* < 0.001); (2) place identity predicts wellbeing (*b* = 0.43, *p* < 0.001); and (3) naturalness predicts wellbeing (*b* = 0.21, *p* < 0.01). It was also reported that naturalness as predictor of wellbeing (“direct effect”) was mediated (“indirect effect”) by place identity (*b* = 0.15, confidence interval (CI) 0.08–0.24, SE = 0.04, *z* = 3.8, *p* < 0.001). The mediation test was performed by computing confidence intervals for the “indirect effect” using bootstrap methods. Concerning the effect size, all (un)standardized confidence intervals contained no-zero point estimates; thus, “we can be confident that the true effects size is different from no effect” (Field, 2013, p. 416).

Accordingly, a significant mediating role of place identity in naturalness-wellbeing relationship was indicated. [*Addendum*: We also performed mediation analyses including emotion and cognition components of place identity, respectively, as mediators. These results were significant (emotion component *b* = 0.22, CI 0.14–0.31, SE = 0.04, *z* = 5.37, *p* < 0.001; and cognition component *b* = 0.07, CI 0.01–0.14, SE = 0.03, *z* = 2.05, *p* = 0.04) as the result at the index level of place identity also was].

## Discussion

The aim of this study was to investigate (1) effects of urban greenery (high vs. low naturalness) on place identity and wellbeing, (2) associations between place identity and wellbeing, and (3) the mediating role of place identity in naturalness-wellbeing relationship. In accord with previous research, we predicted first an effect of high vs. low naturalness on emotional and cognitive components of place identity and wellbeing, second a positive association between the components of place identity and wellbeing, and third a mediating role of place identity in naturalness-wellbeing link.

In line with the first hypothesis, we found that high compared to low naturalness in urban greenery generated a higher place-identity. More precisely, participants living near these milieus were shown to report stronger emotions (attachment/closeness/belonging) and cognitions (remembrance and thinking about and mental travel) in relation to high naturalness greenery. This means that high compared to low naturalness involved more recollections of personal memories and experiences, and affective values, attributed to the high naturalness per se. It was also shown that high naturalness generated higher wellbeing in participants than low naturalness. All this suggests that psychological benefits of urban greenery might increase with naturalness implying that naturalness per se (Ode Sang et al., 2008, 2009; Carrus et al., 2013) might be intertwined with our personal life-story and memory (Knez, 2006; Fivush, 2008; Knez and Eliasson, 2017); thus, our place identity (Marris, 1982; Daniel et al., 2012; Knez, 2014).

Indeed and according to the regression statistics, place identity did significantly predict wellbeing, accounting for 35% of variance explained; however, with a higher increase in wellbeing for every 1-unit increase in the emotional vs. cognitive component. This is in agreement with our second hypothesis, the prediction of Knez (2014), and the findings of Knez and Eliasson (2017), implying that physical places are better remembered if they are “emotionally processed” because emotion may modulate better personal memory (Canli et al., 2000; Phelps, 2006) and pave the way for the cognitive processes of people-place bonding (Knez, 2014).

All this suggests that when visiting high naturalness urban greenery, residents perceive higher levels of wellbeing (Korpela, 1989, 1992; Korpela and Hartig, 1996; Knez, 2006). They do so because they have developed a strong place identity to that type of urban greenery; especially a strong emotional bond to the site (Knez and Eliasson, 2017). This was, finally, supported by the mediation analysis (third hypothesis) showing that a naturalness-wellbeing (Maller et al., 2005; Carrus et al., 2015b) connection might to a certain degree be accounted for by the psychological mechanisms of people-place bonding (Knez, 2014); suggesting that urban greenery might be a part of us (Tuan, 1977; Borrie and Birzell, 2001; Knez, 2006, 2014; Fivush, 2008; McConnell, 2011). This also hints that the self in a self-regulating way (Korpela, 1989, 1992; Korpela and Hartig, 1996; Knez, 2006) might instruct the psychological agent (Bandura, 1991) to enjoy high naturalness in urban greenery; in order to increase wellbeing (Tzoulas et al., 2007; Carrus et al., 2013, 2015a; Tyrväinen et al., 2014) and promote processes of affect-regulation (Parkinson and Totterdell, 1999; Korpela et al., 2008).

What are the practical implications of the results obtained? An increasing body of international research indicates the importance of urban green space to the urban population (Haase et al., 2014). This suggests that urban densification (Westerink and Aalbers, 2013) will not only eat away at green spaces in general but will also erode the smaller urban green spaces that could be of particular importance because of their locality in close relation to people’s homes. Strong personal bonds to particular urban green spaces could be very important to city inhabitants; enhancing wellbeing, as shown in this study. This might partly explain why it is common that plans of densification lead to strong opposition by people living nearby. The loss of smaller woodlands is being compounded by a growing enthusiasm for “improving” the quality of larger public parks; a type of “parkification” (WWF, 2015). This furthermore indicates a reduction of urban green spaces embodying a higher degree of naturalness. Our results highlight the significance of naturalness, meaning that benefits of these types of settings cannot be exchanged by sites that are more intensively managed. In the words of Carrus et al. (2013, p. 234): “Policy makers might want to enhance opportunities for urban residents to encounter settings with high degree of naturalness.”

### Limitations

Two limitations of the present study are appropriate to acknowledge. First, a quasi-experimental design was used for the hypothesis 1 analyses. This design, by definition, lacks random assignment; but, and according to Campbell and Stanley (1963, p. 34); see also Shadish et al. (2002) who disseminated this type of design, there are “many natural social settings in which the researcher can introduce something like experimental design… even though researcher lacks the full control over the scheduling of experimental stimuli.” Accordingly, a quasi-experimental design may be appropriate to use on cross-sectional data (e.g., Knez and Thorsson, 2006, 2008; Marrero and Carballeira, 2010; Ode Sang et al., 2016; Gunnarsson et al., 2017; Hedblom et al., 2017). Second, research in positive psychology has indicated an influence of different demographic variables on wellbeing (e.g., Diener et al., 2002). We did not, however, include any demographic/socioeconomic variable in hypothesis 2 and 3 analyses because the objective of this study was to investigate *general*^1^ relationships between place identity and wellbeing (hypothesis 2) and between naturalness, place identity (as mediator), and wellbeing (hypothesis 3). For that reason, the aim of this article was *not* to investigate what combinations of different types of predictors and controlling variables account for what change in well-being per se, but to explore the *general* associations of (1) place identity → wellbeing and (2) naturalness → place identity → wellbeing, independently of demographic and socioeconomic variables.

## Author Contributions

All authors listed have made a substantial, direct and intellectual contribution to the work, and approved it for publication.

## Conflict of Interest Statement

The authors declare that the research was conducted in the absence of any commercial or financial relationships that could be construed as a potential conflict of interest.

## Footnotes

1 By general we mean a mathematical fact that the greater the magnitude of the slope, the steeper the line and the greater influence of X on Y in a positive, linear, regression function.
